# Impact of white matter hyperintensities on α4β2 nicotinic acetylcholine receptor binding in the human brain

**DOI:** 10.1007/s00259-025-07383-z

**Published:** 2025-06-11

**Authors:** Michael Rullmann, Philipp M. Meyer, Andreas Schildan, Karl-Titus Hoffmann, Osama Sabri, Solveig Tiepolt

**Affiliations:** 1 Department of Nuclear Medicine, University of Leipzig, Liebigstr. 18, 04103 Leipzig, Germany; 2 Department of Neuroradiology, University of Leipzig Medical Center, Leipzig, Germany

**Keywords:** PET, MRI, DTI, α4β2 nicotinic acetylcholine receptor, White matter hyperintensities, Fiber tracking, (-)-[^18^F]Flubatine

## Abstract

**Purpose:**

White matter hyperintensities (WMHs) are commonly observed in aging and neurodegenerative diseases, but their impact on the α4β2 nicotinic acetylcholine receptor (α4β2-nAChR) system remains unclear. This study investigates the relationship between WMHs and gray matter nicotinic signaling, aiming to elucidate potential pathways contributing to neurodegeneration.

**Methods:**

Multimodal imaging data using PET and MR imaging from 39 participants, including 19 healthy controls and 20 patients with Alzheimer’s disease dementia (AD), were analyzed. WMHs were identified on T1-weighted MPRAGE and T2-weighted TSE MR images using advanced segmentation algorithms. Probabilistic fiber tracking was applied to determine WMH-connected gray matter. PET-based total distribution volume (*V*_T_) values of the α4β2-nAChR tracer (-)-[^18^F]Flubatine were compared between WMH-affected and unaffected gray matter regions.

**Results:**

WMH volumes were significantly correlated with age, Fazekas and MMSE scores, but no differences in absolute or relative WMH volumes were observed between healthy controls and patients with AD. PET-based *V*_T_ values in WMH-connected gray matter showed no significant difference from contralateral unaffected regions, regardless of disease status or WMH burden. However, intra-individual differences in *V*_T_ values correlated with Fazekas scores, presumably driven by patients with AD. Pathway-based analyses revealed decreased *V*_T_ values in the medial cholinergic pathway of patients with AD but no significant differences in lateral pathways.

**Conclusion:**

This study shows that WMHs do not significantly alter gray matter nicotinic signaling in directly connected regions. However, the results suggest subtle associations between WMH severity and specific cholinergic pathways, particularly in AD.

**Supplementary Information:**

The online version contains supplementary material available at 10.1007/s00259-025-07383-z.

## Introduction

White matter hyperintensities (WMHs) are regions with abnormal signal intensity in the brain’s white matter, appearing as bright spots on T2-weighted and as darkened areas on T1-weighted MR images (hypointensities) [1, 2]. The biological underpinnings of WMHs are primarily linked to cerebral small vessel disease, which leads to chronic ischemic changes in the white matter [3]. These lesions typically manifest in deep white matter and periventricular areas and reflect a complex interplay of vascular health, white matter integrity and neuronal viability. Specifically, they arise from processes such as myelin loss, gliosis and microvascular dysfunction, leading to localized edema and demyelination [4]. The discovery of WMHs is closely related to the development of MR technology, which allows for detailed visualization of these lesions [5]. WMHs are found to be associated with age [6], vascular risk factors [7, 8] and neurological as well as psychiatric conditions [3, 8, 9]. However, the mechanisms underlying their impact on brain function remain incompletely understood. The cholinergic system, which is critical for maintaining cognitive functions [10, 11], could provide a potential link. Given that disturbances in this system, including α4β2 nicotinic acetylcholine receptor (α4β2-nAChR) availability [12], are hallmarks of aging and Alzheimer’s disease dementia (AD) [13, 14], the interplay between WMHs and cholinergic signaling warrants investigation.

Multimodal imaging using PET and MR technology enables the concurrent assessment of structural abnormalities (e.g. WMHs) and functional alterations (e.g. α4β2-nAChR availability) in the human brain. While it is known that WMHs are associated with cortical function and metabolism [15–17], other studies have also shown a dependency of presence and extent of WMHs on alterations in glucose metabolism [18, 19].

Despite these advances, the impact of WMHs on the α4β2 nicotinic acetylcholine system remains largely unexplored. Given the dependency of WMHs on age and the association of aging with cholinergic system [20, 21] as well as with α4β2-nAChR availability [22, 23], it is crucial to understand whether WMHs contribute to the neurodegenerative loss of nicotinic signals. Such insights are vital for developing interventions aimed at preserving cognitive function in our aging society [24].

To visualize the α4β2-nAChR availability in vivo, the PET tracer (-)-[^18^F]Flubatine was found to improve the specificity and sensitivity of PET imaging to assess α4β2-nAChRs with adequate kinetics for optimal imaging [25, 26]. The PET ligand can serve as a valuable tool for understanding α4β2-nAChRs pathology [12].

This study aims to investigate whether WMHs in the human brain are associated with alterations in nicotinic signals within WMH-connected gray matter. We hypothesize that such changes, if present, would correlate with size of the WMHs and show significant differences in nicotinic binding compared to unaffected WMH-free regions located in the contralateral hemisphere.

## Material and methods

The study cohort is an extension of a previous study [12] comprising 21 patients with mild AD (75 ± 6 years, 5 females) and 20 healthy controls (71 ± 4 years, 12 females). All participants were non-smokers, drug-naїve for cholinesterase inhibitors, free for any centrally acting medication and had no history of neurological or psychiatric disorders other than Alzheimer’s dementia. Diagnosis of AD was made in line with NINCDS-ADRDA criteria. All participants provided written informed consent.

PET data analysis was performed as previously reported [12]. (-)-[^18^F]Flubatine was synthesized in accordance with good manufacturing practice guidelines [27]. PET scans were dynamically acquired following injection of approximately 370 MBq using an ECAT Exact HR + system (Siemens Healthineers, Erlangen, Germany). Dynamic data from 0 to 90 min were used for kinetic analysis. PET data were corrected for scatter, attenuation and radioactive decay, reconstructed using ordered subset expectation maximization with 10 iterations and 16 subsets and subsequently motion-corrected. Logan’s graphical approach, employing the arterial input function, was applied to generate voxel-wise parametric images of total volume of distribution (*V*_T_) [28]. The starting point of t* = 13 min p.i. was chosen based on observed linearity after approximately 10 min in the thalamus as the representative region with the highest α4β2-nAChR density [26]. For each participant approximately 35 arterial blood samples were collected to establish individualized input functions. These included 10–15 samples within the first 3 min p.i., followed by additional samples at 3, 4, 5, 6, 8, 10, 12, 14, 16, 18, 20, 25, 30, 40, 50, 60, 70, 80 and 90 min p.i.. Metabolite analysis was performed on samples taken at 3, 10, 20, 30, 50, 70 and 90 min p.i., revealing high tracer stability with 90% unmetabolised parent compound at 90 min [26]. All samples were centrifuged to separate plasma from the remnants. The radioactivity in plasma aliquots was determined using a Cobra gamma counter (Packard Instrument Company, Meriden, CT, USA) and corrected for decay of ^18^F. Due to the low fraction of metabolites as well as to minimize errors potentially introduced by metabolite correction, kinetic analysis was performed without applying a metabolite correction.

Participants underwent 3 T brain MRI (Magnetom Trio, Siemens Healthineers, Erlangen, Germany) including T1-weighted magnetization prepared rapid gradient echo 3D sequence (MPRAGE, repetition time, TR = 2130 ms, echo time, TE = 3.03 ms, inversion time, TI = 1200 ms, matrix 256 × 256 × 256, pixel bandwidth 130 Hz), T2-weighted turbo spin echo (TSE, TR = 6000 ms, TE = 103 ms, pixel bandwidth 220 Hz, slice thickness = 2 mm, FOV = 256 × 256 mm^2^, 256 × 256 image matrix) and diffusion-weighted imaging (DWI) with 65 axial slices with 2 mm thickness (no gap) covering the entire brain with a twice-refocused spin echo echo-planar-imaging sequence. We used the following parameters: TE = 116 ms, TR = 15.9 s, α = 90°, bandwidth = 1000 Hz/pixel, FOV = 256 × 256 mm^2^, 128 × 128 image matrix. Diffusion-weighted data were acquired with 30 diffusion-encoding gradient directions (diffusion tensor imaging-DTI) and a b-value of 1000 s/mm^2^. In addition, one volume was recorded without diffusion weighting (b-value 0 s/mm^2^) as anatomical reference for offline motion correction. For one patient, the DWI measurements were not performed. Average time delay between PET and MRI acquisition was 10 days (ranging from 1 to 34 days).

To identify WMHs we applied two different toolboxes qualified to segment T1-weighted MRI’s surrogate of WMHs, white matter hypointensities: (1) Sequence Adaptive Segmentation (SAMSEG [29]) together with T1-MPRAGE and T2-TSE images as part of the FreeSurfer software suite and (2) CAT12 [30] a toolbox for SPM12 [31] to segment T1-MPRAGE images. To build the final WMH mask, we first computed the intersection of both WMH masks (logical AND operator), identified the ventricle (based on CAT12 segmentation) and dilated the ventricle mask by 5 mm, which was then used to remove any potential periventricular hyperintensities. In one healthy control, there were no WMHs left after removal. We investigated the absolute WMH surrogate volume (hereinafter referred to as WMH volume) as well as the WMH volume relative to the individual total intracranial volume.

Evaluation of WMHs followed the well-established Fazekas scoring scheme [32] with values between zero and three. In this study, we focus on the Fazekas score in the deep white matter, as cholinergic pathways are thought to be predominantly localized there compared to the periventricular white matter [33].

For DWI preprocessing we used FMRIB's Diffusion Toolbox as part of the FSL pipeline [34] including the tool eddy [35] for correction of eddy currents and movements, the tool bedpostx for estimation of multiple fiber orientations using the GPU implementation [36] of Bayesian Estimation of Diffusion Parameters Obtained using Sampling Techniques for modelling Crossing Fibers [37] and the tool probtrackx for probabilistic tracking with crossing fibers (GPU implementation [38]). All tools were applied with their default values [39]. For probabilistic tractography analysis we applied the WMH mask as region of interest. This results in a 3D volume containing the output connectivity distribution to the WMH mask. One of the resulting parameter is the total amount of generated tracts (waytotal), which depends on the size of the seed mask (WMH volume), hence we also investigated the amount of tracts relative to the individual WMH volume.

We used the fiber tracking mask to identify all gray matter areas which are connected to the related WMHs. Therefore, we thresholded the fiber tracking mask by 5% to remove the weakest, outlier sensitive connections and subsequently computed the intersection with the gray matter segmentation to constrain the mask to the gray matter areas of the brain. We then averaged the PET signal over those WMH connected gray matter areas. To determine unaffected regions as a control condition, we flipped the outlier-removed fiber tracking mask to the contralateral hemisphere, constrained the mask to gray matter areas only and removed any overlays with the non-flipped mask. To investigate a potential volume effect of the identified masks, we analyzed enlarged gray matter masks (dilated by 4 mm) in an additional analysis. Gray matter masking and flipping was performed in MNI space, while spatial normalization was determined based on the T1-weighted data in SPM12.

In addition, we identified trajectories of cholinergic pathways [33] in the JHU DTI-based white matter atlas [40]. Within the three pathways, medial and lateral (capsular and perisylvian division) pathways, we calculated the WMH volumes and the PET signal at the intersection of the pathways with the gray matter.

### Statistics

To investigate potential dependencies of WMH related PET signal alterations we divided our cohort according to disease stage (healthy controls vs. patients with Alzheimer’s disease), amount of WMH (median split based on the relative WMH volume with total intracranial volume as reference) and Fazekas score in the deep white matter (four groups with scores of zero, one, two and three). Comparisons between the different groups were performed using two-sampled Student’s t test, Wilcoxon rank sum test or ANOVA (with Tukey–Kramer post-hoc test). Within-subject analyses were performed using a paired t test. Intra-individually differences were computed by subtracting contralateral unaffected from the affected values. Group differences in count data (disease status, sex) were tested using Fisher’s exact test. Correlation were calculated using Spearman correlation coefficient. All calculations were computed in Matlab (v9.6, The Mathworks Inc., Natick, Massachusetts, USA). Significance was assumed at *p* < 0.05 level. In addition, we corrected the results for multiple comparisons by applying Bonferroni correction. The tract-based results were corrected for the number of groups (*n* = 3 for two-sampled group comparisons in Table 1; *n* = 9 for within-subject paired t test in Fig. 3A; *n* = 5 for correlation analysis in Fig. 3B and C). The atlas-based results were corrected for the number of investigated pathways (*n* = 3).

**Table 1:** Comparison between healthy controls (HC) and patients with Alzheimer’s disease (AD), between participants with less and more white matter hyperintensities (WMHs, median splitted) and between participants stratified by their Fazekas scores. Results include demographic parameters (sex, age, MMSE score, Fazekas score in the deep white matter) as well as DTI fiber tracking-based parameters (absolute and relative WMH volume, absolute and relative total amount of generated tracts (waytotal), PET-based distribution volume (*V*_T_) values in WMH-connected gray matter)

Parameter	HC	AD	*p*	< WMH	> WMH	*p*	Fazekas score				*p*
							0	1	2	3	
Disease state [HC/AD]	19/0	0/20	**-**	13/7	6/13	0.061^$^	6/4	12/7	1/8	0/1	**0.028**^$^
Sex [male/female]	8/11	15/5	0.054^$^	11/9	12/7	0.75^$^	8/2	8/11	6/3	1/0	0.16^$^
Age [years]	70.6 ± 4.4	74.9 ± 6.1	**0.019**^‡^	70.1 ± 5.8	75.6 ± 4.1	0.0016*^‡^	68 ± 5.2	72.5 ± 4.5	78.3 ± 3.9	76.6 ± 0	2.8e-4*^§^
MMSE score	28.6 ± 0.69	23.6 ± 2.6	2e-9*^‡^	27.4 ± 2.2	24.8 ± 3.5	0.011*^‡^	27.6 ± 1.6	26.7 ± 3.1	23.7 ± 3.3	23.0 ± 0	**0.021**^§^
Fazekas score	0.74 ± 0.56	1.3 ± 0.86	**0.028**^**†**^	0.55 ± 0.51	1.5 ± 0.7	7.9e-5*^†^	-	-	-	-	-
Absolute WMH volume [mm^3^]	736 ± 1446	1295 ± 1407	0.23^‡^	89 ± 83	2006 ± 1534	2.3e-5*^‡^	182 ± 272	705 ± 1188	2645 ± 1562	882 ± 0	2.5e-4*^§^
Relative WMH volume [%]	0.05 ± 0.1	0.09 ± 0.094	0.22^‡^	0.006 ± 0.0052	0.14 ± 0.11	2e-6*^‡^	0.01 ± 0.02	0.05 ± 0.08	0.18 ± 0.1	0.07 ± 0	2.2e-4*^§^
Waytotal [*N*]	301316 ± 604078	547750 ± 593015	0.21^‡^	33250 ± 32658	842895 ± 642667	2e-6*^‡^	69000 ± 107103	302895 ± 510036	1098333 ± 653806	350000 ± 0	3.4e-4*^§^
Waytotal/WMH volume [N/mm^3^]	364 ± 98	477 ± 290	0.11^‡^	423 ± 310	420 ± 64	0.97^‡^	488 ± 428	387 ± 93	424 ± 50	397 ± 0	0.73^§^
(-)-[^18^F]Flubatine [*V*_T_]	8.6 ± 0.83	8.7 ± 1.7	0.88^‡^	8.4 ± 0.79	8.9 ± 1.7	0.21^‡^	8.4 ± 0.79	8.5 ± 0.83	9.3 ± 2.4	8.0 ± 0	0.37^§^

^$^Fisher exact test; ^‡^Student’s t test; ^†^Wilcoxon rank sum test; ^§^ANOVA; significance marked in **bold**; *P values remain significant after Bonferroni correction for multiple comparisons

## Results

**Fig. 1:**
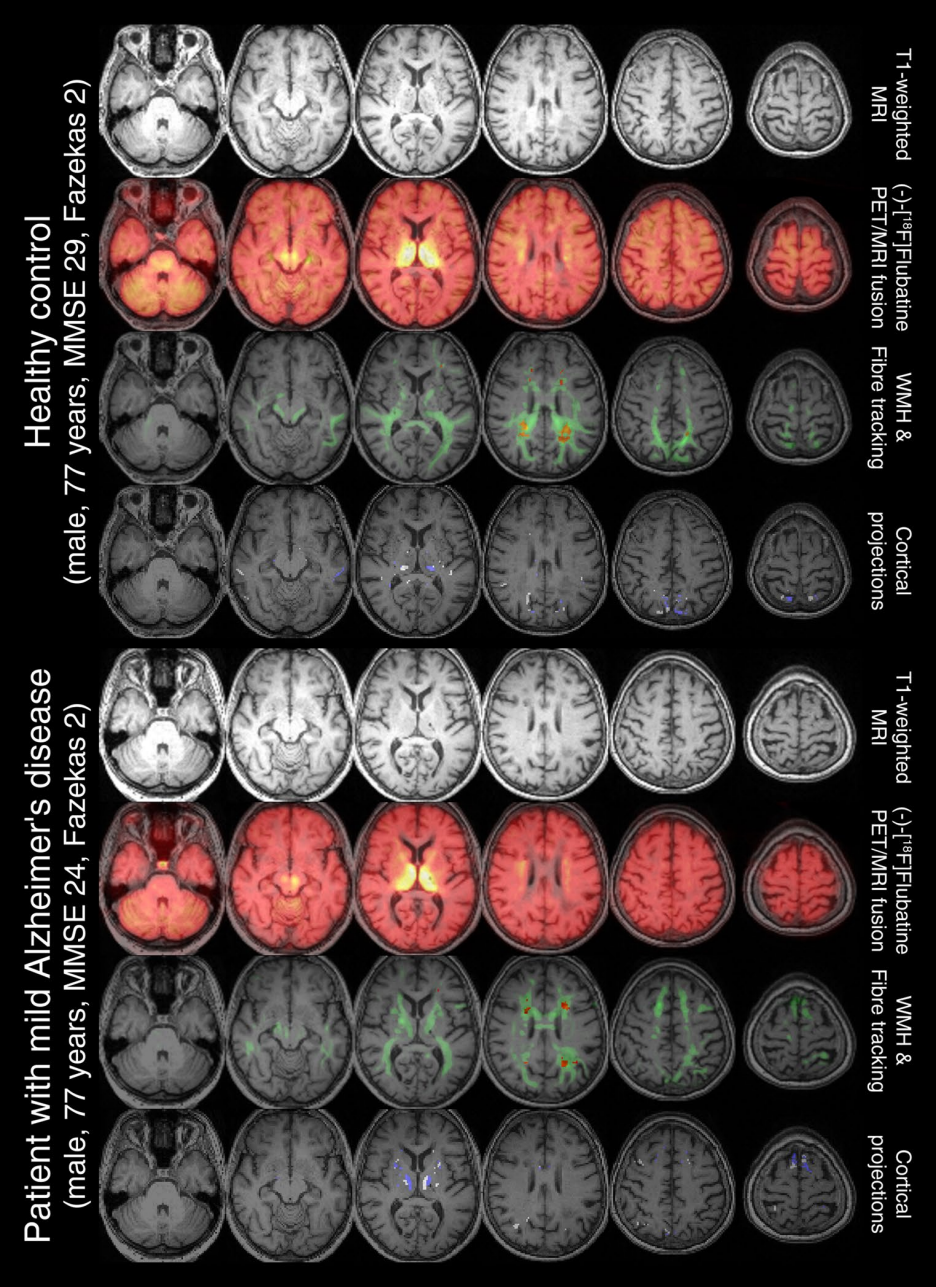
Two cases with a Fazekas score of 2 are presented showcasing its different imaging and processing modalities. For each case, the first row displays T1-weighted MR images, while the second row introduces a fused view of (-)-[^18^F]Flubatine PET images. The third row overlays the identified white matter hyperintensities (WMH) in red along with he corresponding fiber tracts in green, as determined by fiber tracking. The fourth row highlights the WMH-connected gray matter (blue) and their unaffected contralateral counterparts (white), which were used to extract PET-based distribution volumes. MMSE: mini-mental state examination

The original study cohort consisted of 20 healthy controls and 21 patients with mild AD. We excluded one patient without DWI measurements and one healthy control without WMHs. Thus, our current study included data of 19 healthy controls (11 females, age: 70.6 ± 4.4 years, MMSE: 28.6 ± 0.7) and 20 patients with early AD (5 females, age: 74.9 ± 6.1 years, MMSE: 23.6 ± 2.6). Exemplary cases are shown in Fig. 1 including axial views of T1 weighted MR image, WMH segmentation, connectivity distribution of fiber tracking and WMH-connected gray matter as well as areas of the control condition (WMH-free regions located in the contralateral hemisphere).

**Fig. 2:**
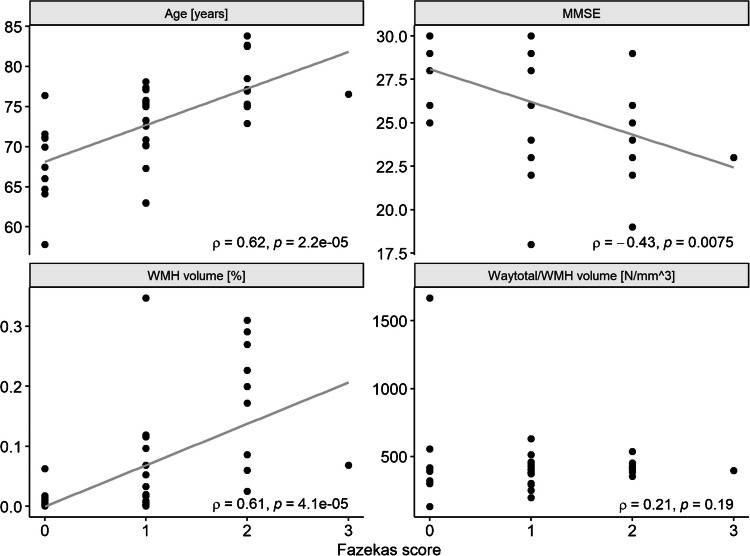
Associations of Fazekas score in the deep white matter with age, MMSE, relative white matter hyperintensity (WMH) volumes and relative amount of generated tracts. MMSE: mini-mental state examination; *ρ*: Spearman correlation coefficient

We observed higher Fazekas scores, higher age and lower mini-mental state examination (MMSE) values in patients with AD when compared to healthy controls (*p* < 0.05, Table 1). No differences in WMH volumes between both groups were found, neither in absolute values (*p* = 0.23), nor in relation to the individual total intracranial volume (*p* = 0.22, Table 1). The total amount of generated tracts (parameter: waytotal), absolutely or in relation to the WMH volume, does not differ significantly, either (see Table 1). We found significant correlations of age, MMSE scores and relative WMH volume with the Fazekas score evaluated in the deep white matter (Fig. 2). The atlas-based analysis of WMH volumes showed significant differences in all three cholinergic pathways with higher (absolute and relative) volumes in patients with AD when compared to healthy controls (see Table 2).

**Table 2:** Atlas-based results between healthy controls (HC) and patients with Alzheimer’s disease (AD)

Parameter	HC	AD	*p*^‡^
WMH volume [mm^3^]			
Medial pathway Lateral pathway: capsular division Lateral pathway: perisylvian division	494 ± 66 651 ± 75 547 ± 65	597 ± 89 711 ± 86 563 ± 55	2.4e-4* **0.027** 0.42
WMH volume [%]			
Medial pathway Lateral pathway: capsular division Lateral pathway: perisylvian division	0.033 ± 0.0049 0.043 ± 0.0068 0.036 ± 0.0058	0.043 ± 0.0077 0.051 ± 0.0089 0.041 ± 0.0064	2.1e-5* 0.0035* **0.041**
(-)-[^18^F]Flubatine [*V*_T_]			
Medial pathway ∩ GM Lateral pathway: capsular division ∩ GM Lateral pathway: perisylvian division ∩ GM	6.2 ± 0.56 9.5 ± 0.6 9.9 ± 0.63	5.8 ± 0.78 9.6 ± 1.0 10.1 ± 1.1	**0.048** 0.99 0.41

∩: intersection of masks; *GM* gray matter; ^‡^Student’s t test; significance marked in bold; *P values remain significant after Bonferroni correction for multiple comparisons

After performing a median split (based on the relative WMH volume), the two groups, of course, differed significantly in WMH volumes (*p* < 0.001), as well as in age (*p* = 0.0016), MMSE scores (*p* = 0.01) and Fazekas score (*p* < 0.001), but not in disease status (*p* = 0.06, Table 1).

Stratifying the cohort according to their deep white matter Fazekas score in four groups, an ANOVA revealed differences in the WMH volumes (absolute: F(3) = 9.09, *p* < 0.001, relative: F(3) = 9.26, *p* < 0.001), whereas Tukey–Kramer post-hoc tests showed, that participants with a Fazekas score of 2 differed significantly from that with scores of 0 and 1. The groups also varied in age (F(3) = 8.3, *p* < 0.001) and disease status (*p* = 0.03, Table 1).

**Fig. 3:**
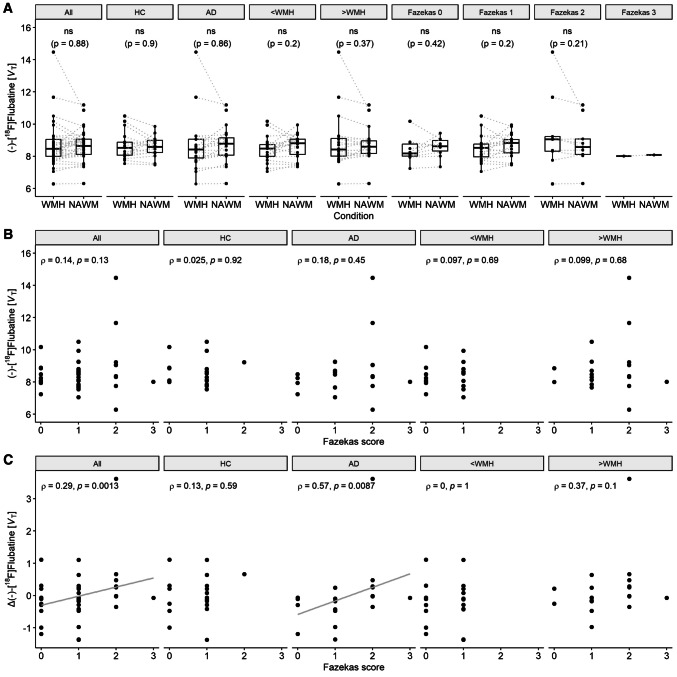
**A** Distribution of pairwise PET-based distribution volume (*V*_T_) values in white-matter-hyperintensity-connected (WMH) and unaffected (NAWM) gray matter. **B** Association between PET-based *V*_T_ values in WMH-connected gray matter and **C** intra-individual difference of *V*_T_ values (Δ) between affected and unaffected regions in relation to the Fazekas score. Data is shown for healthy controls (HC) and patients with Alzheimer’s disease (AD), groups with less (< WMH) and more (> WMH) volume (based on median split of relative WMH volumes) and grouped by Fazekas score (only subplot (A)). ρ: Spearman correlation coefficient. Results remain significant after Bonferroni correction for multiple comparisons

The PET-based distribution volume in the WMH-connected gray matter did not differ significantly compared to their contralateral unaffected counterparts (Fig. 3A), neither in the whole group nor in subgroups of healthy controls, patients with AD, median-splitted groups with less or more WMH volume or groups categorized by their Fazekas score (Supplemental Table 2). In all subgroup analyses, including disease status, WMH volume and Fazekas score categories, no significant group differences were found in WMH-connected *V*_T_ values (see Table 1). Investigating the same effect applying enlarged masks showed no significant differences (Supplemental Fig. 1), either.

There was no significant correlation of the affected distribution volume with the Fazekas score (Fig. 3B). Inspecting the intra-individual difference, i.e. the difference between affected and unaffected values, there was a significant association with the Fazekas score (*ρ* = 0.29, *p* = 0.001, remains significant after Bonferroni correction), which seems to be driven by the group of patients with AD (*ρ* = 0.57, *p* = 0.009, remains significant after Bonferroni correction). No other subgroup presented a significant correlation (Fig. 3C).

While we found a difference in the PET-based *V*_T_ signals at the intersection with the medial pathway when comparing healthy controls with patients with AD (6.2 ± 0.6 vs. 5.8 ± 0.8 *V*_T_, *p* = 0.048), we found no significant effects in both lateral pathways (see Table 2). That significant finding vanishes after Bonferroni correction and when applying enlarged masks (Supplemental Table 3). The median splitted groups showed no significant effects in the PET signal intersected with one the three pathways (medial: 6.1 ± 0.7 vs 5.9 ± 0.7, *p* = 0.51; lateral at capsular division: 9.7 ± 0.8 vs. 9.4 ± 0.8, *p* = 0.27; lateral at perisylvian division: 10.2 ± 0.9 vs. 9.9 ± 0.9, *p* = 0.25). Subgroup analysis based on Fazekas scores showed no significant differences of the atlas-based pathways intersected PET signal (medial: F(3) = 1.49, *p* = 0.23; lateral at capsular division: F(3) = 1.13, *p* = 0.35; lateral at perisylvian division: F(3) = 0.71, *p* = 0.55).

## Discussion

This study investigated the relationship between WMHs and gray matter nicotinic binding in WMH-connected regions using multimodal (-)-[^18^F]Flubatine PET and MR imaging, considering factors such as disease status and WMH burden, as assessed by volume or Fazekas score. Our findings suggest that the presence and extent of WMHs, particularly in deep white matter, do not significantly alter gray matter nicotinic binding when compared to contralateral unaffected regions. This observation is consistent across various group stratifications including disease status, WMH volume and Fazekas score.

The lack of significant differences in α4β2-nAChR availability between WMH-affected gray matter and their contralateral unaffected counterparts suggests that WMHs, despite their associations with aging [41, 42] and neurodegenerative processes [43, 44], may not directly affect cholinergic function in the early or intermediate stages of WMH progression. Previous studies have shown that aging impacts the cholinergic system [22, 45] including a decline in α4β2-nAChR availability [12], but our results indicate that these changes may not be exacerbated by WMH-related disruptions in connectivity. This lack of significant variation held true across various subgroup analyses including disease status, WMH volume and Fazekas score categories.

Interestingly, we observed significant correlations between Fazekas scores and WMH volume, age and cognitive decline, as measured by MMSE. These results align with existing literature that demonstrates a link between WMHs, cognitive impairment and vascular contributions to neurodegeneration [46–50]. However, the absence of direct effects on nicotinic signals indicates a potentially preserved function of the cholinergic system within WMH-connected regions, which could have implications for therapeutic strategies aimed at preserving cognitive function in aging populations [51, 52].

While we did not observe a significant group-level difference in PET-based total volume of distribution between affected WMH-connected and contralateral unaffected regions, we did find a significant positive correlation between the within-subject *V*_T_ difference and overall WMH burden, as measured by the Fazekas score. This correlation suggests a link between regional nicotinic alterations and increasing lesion load. However, a correlation does not provide information on causality. But it highlights the importance of considering individual variability in WMH burden when assessing neurochemical effects like changes in nicotinic acetylcholine receptor binding. The identification of WMH-connected cortical regions was based on a robust segmentation approach using two independent algorithms and tractography masks were thresholded to reduce the influence of weak or spurious connections. However, we acknowledge that the resulting cortical masks are highly dependent on WMH volume: in participants with low WMH burden, these masks are smaller and thus more sensitive to local noise and outliers. Despite this, the use of contralateral regions as intra-individual controls helps to mitigate anatomical variability and enhance interpretability. Future studies with larger samples, more severe disease stage and higher WMH loads are needed to further validate these findings and improve the stability of regional measurements.

Further exploration revealed significant differences in (-)-[^18^F]Flubatine PET-based α4β2-nAChR signals along the medial cholinergic pathway in patients with AD compared to healthy controls. This finding aligns with previous studies that identify the medial pathway as a critical structure impacted by AD [53, 54] and suggests that while WMHs may be spatially associated with cholinergic pathways, their impact on α4β2 nicotinic receptor availability remains limited and disease-specific, especially since the effect vanished after multiple comparison correction. Atlas-based analyses confirmed the tract-based results of higher WMH burden in patients with AD when compared to healthy controls.

The cholinergic hypothesis suggests that the degeneration of cholinergic neurons, the resulting decrease in acetylcholine levels and impairments in cholinergic signaling contribute significantly to the cognitive deficits observed in individuals affected by AD [55, 56]. The pathological mechanisms underlying cholinergic dysfunction in AD include the accumulation of β-amyloid plaques and neurofibrillary tangles, which are believed to trigger cytotoxic processes leading to cholinergic neuron degeneration [14, 57]. In our study cohort including patients with mild AD we found no clear evidence that vascular alterations, as measured by WMHs, significantly impacts cholinergic integrity.

While our findings highlight the complexity of WMH-related changes in the cholinergic system, further research is needed to clarify the mechanistic pathways linking WMHs, cholinergic degeneration and cognitive impairment. Longitudinal studies involving larger cohorts could provide deeper insights into the temporal dynamics of these interactions. Furthermore, research into interventions targeting WMH-associated cholinergic dysfunction could open new avenues for preserving cognitive function in our aging society. This underscores the importance of integrating multimodal imaging to decipher the interplay between structural and functional brain changes.

### Limitations

The current standard imaging sequence to detect WMH is T2 weighted fluid-attenuated inversion recovery (FLAIR), which uses a long inversion time to additionally suppress CSF signal [58]. However, the MRI protocol in this study did not include a FLAIR sequence. Instead, we utilized T1-weighted MPRAGE and T2-weighted TSE sequences in combination with advanced algorithms [29, 30], which have demonstrated robust performance to generate a reliable surrogate. To minimize potential segmentation errors from individual methods, the results of both approaches were integrated.

Another potential limitation is the theoretical presence of partial volume effects, which could influence PET signal measurements. However, this effect is expected to be minimal, as the gray matter regions analyzed exhibit relatively homogeneous tracer uptake (see Fig. 1), reducing the likelihood of significant spillover artifacts. In addition, the theoretical effect should be approximately equal in both the affected and unaffected gray matter regions due to its left/right flipped origin. Furthermore, our previous study investigating the same dataset reported no significant partial volume effects [12].

While our statistical analyses observed no significant differences of nicotinic signals when compared to contralateral unaffected regions, it cannot be assumed that there was no impact. Failing to reject the null hypothesis means there is currently not enough evidence to support the alternative hypothesis. However, it does not imply that the null hypothesis is true. Reasons could be e.g. a too small sample size to detect the effect or a high variability in the data (the effect exists, but noise swamped the effect) [59]. A post-hoc sensitivity power analysis (G*Power, University of Kiel, Germany) reveals a minimal detectable effect size of 0.46 (paired t test at 0.8 power with *n* = 39), i.e. our test could reasonably exclude a medium effect size.

## Conclusion

In conclusion, our results highlight the complex interplay between WMHs, cholinergic pathways and gray matter α4β2-nAChR signals. While WMHs are associated with aging and cognitive decline, their impact on nicotinic function appears limited. These findings contribute to the understanding of WMH-induced brain changes and emphasize the need for further research into compensatory mechanisms and into their functional consequences in aging and neurodegenerative diseases.

## Supplementary Information

Below is the link to the electronic supplementary material.

Supplementary file1 (DOCX 113 KB)

## Data Availability

The datasets generated and analysed during the current study are available from the corresponding author on reasonable request. The data are not publicly available due to their containing information that could compromise the privacy of the participants.
