# Supplementary material for: Impact of white matter hyperintensities on α4β2 nicotinic acetylcholine receptor binding in the human brain
Source: Eur J Nucl Med Mol Imaging. 2025 Jun 11;52(13):5113–23. doi: 10.1007/s00259-025-07383-z (PMC12589245; doi:10.1007/s00259-025-07383-z)
Supplement: Supplementary file 1 — Supplementary file1 (DOCX 113 KB) [file 259_2025_7383_MOESM1_ESM.docx]

# Impact of white matter hyperintensities on α4β2 nicotinic acetylcholine receptor binding in the human brain

Michael Rullmann^1^, Philipp Meyer^1^, Andreas Schildan^1^, Karl-Titus Hoffmann^2^, Osama Sabri^1*^, Solveig Tiepolt^1*^

^1^ Department of Nuclear Medicine, University of Leipzig, Leipzig, Germany

^2^ Department of Neuroradiology, University of Leipzig Medical Center, Leipzig, Germany

* Authors contributed equally to this work

## Supplementary Material

Supplemental Figure 1: **(A)** Distribution of pairwise PET-based distribution volume (*V*_T_) values in white-matter-hyperintensity-connected (WMH) and unaffected (NAWM) gray matter applying enlarged masks (dilated by 4 mm within gray matter region). **(B)** Associations of intra-individual difference of *V*_T_ values (Δ) between affected and unaffected regions with enlarged masks in relation to the Fazekas score. Data is shown for healthy controls (HC) and patients with Alzheimer’s disease (AD), groups with less (<WMH) and more (>WMH) volume (based on median split of relative WMH volumes) and grouped by Fazekas score. ρ: Spearman correlation coefficient.


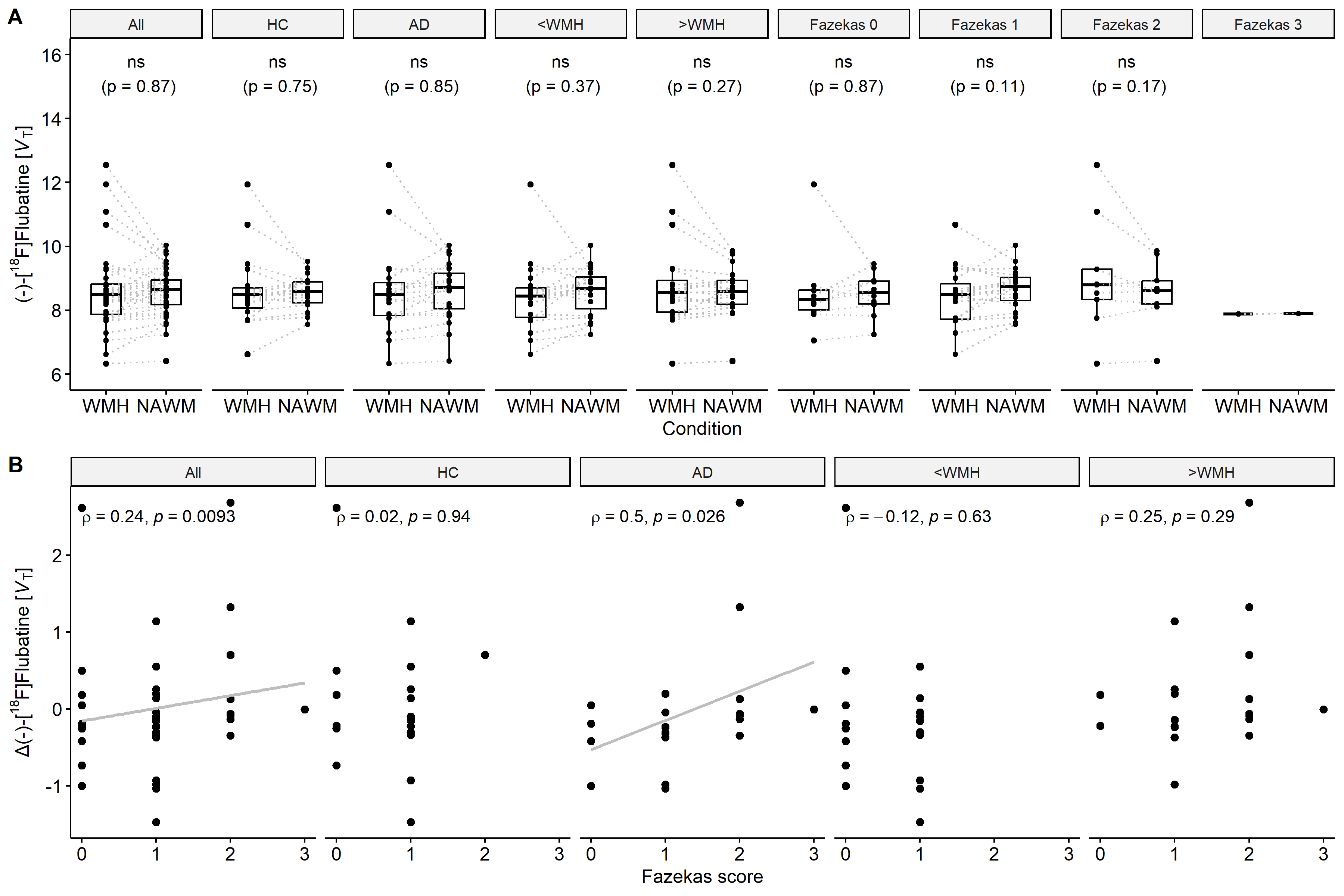


Supplemental Table 1: Further subgroup analyses in addition to the results shown in Table 1.

Comparison between participants with less and more white matter hyperintensities (WMHs, median splitted) grouped by their disease status (healthy controls, HC and patients with Alzheimer’s disease, AD) and between participants stratified by their Fazekas scores. Results include demographic parameters (sex, age, MMSE score, Fazekas score in the deep white matter) as well as DTI fiber tracking-based parameters (absolute and relative WMH volume, absolute and relative total amount of generated tracts (waytotal), PET-based distribution volume (DV) values in WMH-connected gray matter).

| **Parameter** | **<WMH** | **>WMH** | **p** | **Fazekas score** | | **p** | **Fazekas score (AD only)** | | **p** |
| --- | --- | --- | --- | --- | --- | --- | --- | --- | --- |
|  |  |  |  | **0/1** | **2/3** |  | **1** | **2** |  |
| Disease state [HC/AD] | HC: 13 AD: 7 | HC: 6 AD: 13 | HC: - AD: - | 18/11 | 1/9 | **0.008**^$^ | 0/7 | 0/8 | - |
| Sex [male/female] | HC: 5/8 AD: 6/1 | HC: 3/3 AD: 9/4 | HC: 1^$^ AD: 0.61^$^ | 16/13 | 7/3 | 0.48^$^ | 5/2 | 5/3 | 1^$^ |
| Age [years] | HC: 69.4 ± 4.7 AD: 71.4 ± 7.8 | HC: 73.3 ± 2.6 AD: 76.7 ± 4.3 | HC: 0.08^‡^ AD: 0.06^‡^ | 70 ± 5.2 | 78 ± 3.7 | **<0.001**^‡^ | 75.1 ± 3.3 | 78.5 ± 4.1 | 0.11^‡^ |
| MMSE score | HC: 28.6 ± 0.8 AD: 24.7 ± 1.8 | HC: 28.5 ± 0.5 AD:23.2 ± 2.9 | HC: 0.75^‡^ AD: 0.25^‡^ | 27 ± 2.7 | 23.6 ± 3.1 | **0.002**^‡^ | 23.6 ± 2.9 | 23 ± 2.8 | 0.71^‡^ |
| Fazekas score | HC: 0.61 ± 0.5 AD: 0.43 ± 0.5 | HC: 1 ± 0.6 AD: 1.8 ± 0.6 | HC: 0.35^†^ AD: **0.001^†^** | - | - | - | - | - | - |
| Absolute WMH volume  [mm^3^] | HC: 101 ± 98 AD: 68 ± 41 | HC: 2112 ± 2044 AD: 1957 ± 1335 | HC: **0.002**^‡^ AD: **0.002**^‡^ | 524 ± 997 | 2469 ± 1575 | **<0.001**^‡^ | 764 ± 761 | 2421 ± 1508 | **0.02**^‡^ |
| Relative WMH volume  [%] | HC: 0.006 ± 0.006 AD: 0.005 ± 0.003 | HC: 0.15 ± 0.15 AD: 0.13 ± 0.09 | HC: **0.002**^‡^ AD: **0.001**^‡^ | 0.04 ± 0.07 | 0.17 ± 0.1 | **<0.001**^‡^ | 0.05 ± 0.05 | 0.17 ± 0.1 | **0.02**^‡^ |
| Waytotal [N] | HC: 36923 ± 39557 AD: 26429 ± 12817 | HC: 874167 ± 856962 AD: 828462 ± 559305 | HC: **0.002**^‡^ AD: **0.002**^‡^ | 222241 ± 428624 | 1023500 ± 660278 | **<0.001**^‡^ | 357143 ± 407440 | 998125 ± 620679 | **0.04**^‡^ |
| Waytotal / WMH volume [N/mm^3^] | HC: 346 ± 115 AD: 566 ± 491 | HC: 401 ± 24 AD: 429 ± 75 | HC: 0.27^‡^ AD: 0.33^‡^ | 422 ± 259 | 421 ± 48 | 0.99^‡^ | 428 ± 108 | 423 ± 54 | 0.92^‡^ |
| (-)-[^18^F]Flubatine [DV] | HC: 8.5 ± 0.8 AD: 8.1 ± 0.8 | HC: 8.8 ± 1.0 AD: 9.0 ± 2.0 | HC: 0.53^‡^ AD: 0.29^‡^ | 8.5 ± 0.8 | 9.2 ± 2.3 | 0.13^‡^ | 8.4 ± 0.8 | 9.4 ± 2.6 | 0.37^‡^ |

^$^Fisher exact test; ^‡^Student’s t test; ^†^Wilcoxon rank sum test; ^§^ANOVA; significance marked in **bold**

Supplemental Table 2: Intra-individual comparison of (-)-[^18^F]Flubatine *V*_T_ values between affected and unaffected regions.

| Group | Affected regions [mean ± SD] | Unaffected regions [mean ± SD] | Δ*V*_T_ [mean ± SD] | p^‡^ |
| --- | --- | --- | --- | --- |
| All | 8.7 ± 1.4 | 8.7 ± 0.9 | 0.01 ± 0.82 | 0.88 |
| Healthy controls | 8.6 ± 0.8 | 8.6 ± 0.6 | 0.02 ± 0.61 | 0.9 |
| Patients with Alzheimer’s disease | 8.7 ± 1.7 | 8.7 ± 1.1 | -0.04 ± 0.98 | 0.86 |
| <WMH | 8.4 ± 0.8 | 8.6 ± 0.7 | -0.21 ± 0.7 | 0.2 |
| >WMH | 8.9 ± 1.7 | 8.7 ± 1.1 | 0.2 ± 0.9 | 0.37 |
| Fazekas score 0 | 8.4 ± 0.8 | 8.6 ± 0.6 | -0.17 ± 0.65 | 0.42 |
| Fazekas score 1 | 8.5 ± 0.8 | 8.7 ± 0.7 | -0.18 ± 0.61 | 0.2 |
| Fazekas score 2 | 9.4 ± 2.4 | 8.8 ± 1.5 | 0.54 ± 1.19 | 0.21 |
| Fazekas score 3 | 8.0 ± 0 | 8.1 ± 0 | -0.1 ± 0 | - |

^‡^paired t test

Supplemental Table 3: Atlas-based results between healthy controls (HC) and patients with Alzheimer’s disease (AD) with enlarged masks (dilated by 4mm within gray matter region).

| Parameter | HC | AD | p^‡^ |
| --- | --- | --- | --- |
| (-)-[^18^F]Flubatine [*V*_T_]  Medial pathway ∩ GM  Lateral pathway: capsular division ∩ GM  Lateral pathway: perisylvian division ∩ GM | 8.8 ± 0.53  8.5 ± 0.48  8.6 ± 0.52 | 8.3 ± 1.1  8.5 ± 1.1  8.7 ± 1.1 | 0.35  0.97  0.9 |

∩: intersection of masks; GM: gray matter; ^‡^Student’s t test;
